# Target area treatment ratio of varied lesions in the cultured pure melanocyte transplantation repigmentation of vitiligo: A retrospective study

**DOI:** 10.1111/1346-8138.17320

**Published:** 2024-06-19

**Authors:** Fuquan Lin, Yunxia Wang, Yujie Zheng, Weisong Hong, Lifang Fu, Miaoni Zhou, Aie Xu

**Affiliations:** ^1^ Department of Dermatology The First Affiliated Hospital of Anhui Medical University Hefei China; ^2^ Department of Dermatology Hangzhou Third People's Hospital Hangzhou China; ^3^ Hangzhou Third People's Hospital Affiliated to the Zhejiang Chinese Medical University Hangzhou China

**Keywords:** melanocytes, regional, transplanation, treatment effectiveness, vitiligo

## Abstract

Autologous cultured pure melanocyte transplantation (CMT) can be utilized to treat stable vitiligo cases, but clinical data are insufficient to improve its efficacy. To evaluate the influence of various factors on the therapeutic effect of CMT, this single‐center retrospective study enrolled stable vitiligo patients who underwent CMT between 2009 and 2020. Univariate and multivariable analysis were used to determine the factors affecting the outcome of repigmentation. The study included 491 patients with long‐term follow‐up data (6–120 months). It was found that 69.7% of patients achieved an excellent re‐color effect and 18.4% achieved a good re‐color effect. There were statistically significant differences in pigmentation between patients with stable disease course, vitiligo type, and lesion site. Overall, a significant positive correlation between the target area treatment ratio of varied lesions and the percentage of repigmentation was found. CMT is effective and well tolerated in the treatment of stable vitiligo. Various factors, especially the target area treatment ratio of varied lesions, should be carefully assessed before using CMT. As the target area treatment ratio of varied lesions could further improve the post‐operative repigmentation other than type of vitiligo. This clinic trial was approved by Hangzhou Third People's Hospital (number 2023KA015, national clinical record number MR‐33‐23‐034502).

## 1 INTRODUCTION

Vitiligo is a common hypopigmentary dermatosis characterized by patches of depigmentation affecting approximately 0.1%–2.0% of the global population.^1^, ^2^, ^3^, ^4^, ^5^ Although the disease does not directly cause physical impairment, it influences patients' quality of life and psychological health.^6^, ^7^, ^8^

Vitiligo treatment remains a challenge for dermatologists and is often unsatisfactory. Many options have been proposed and are currently available, including traditional pharmacological approaches such as topical and systemic corticosteroids, topical calcineurin phosphatase inhibitors, vitamin D derivatives, and phototherapy.^3^, ^4^, ^9^, ^10^, ^11^, ^12^ Surgical treatment is a valuable alternative for patients with stable vitiligo who are non‐responsive to traditional medication. In our previous studies, autologous cultured pure melanocyte transplantation (CMT) in patients with stable vitiligo achieved favorable outcomes.^13^, ^14^, ^15^ Guidelines recommend using combination medical therapy that includes at least one light therapy for the combined treatment of vitiligo. However, large‐scale stable surgery is a valuable alternative in segmental and non‐segmental vitiligo (SV/NSV).^16^

Lerner first described autologous CMT for the treatment of vitiligo in 1987.^17^ Since then, several studies on this technique have been reported, including Olsen's clinical studies.^18^ ^,19^ Extending cell populations in vitro provides more melanocytes for transplantation, therefore cells from a small piece of donor skin can be used to treat large vitiliginous lesions (up to 500 cm^2^) with an expansion ratio of >1:10.^18^ ^−22^

Several reports have described the effectiveness of CMT in treating stable vitiligo, but only a few enrolled a large sample size, and few analyzed the relevant factors influencing the results of transplantation. Furthermore, although the disease site and therapeutic responses to transplantation might be closely related, few studies have reported the relationship between the site and effectiveness of transplantation.

In the present study, we applied the CMT to 491 patients with vitiligo. Patients were followed up for up to 12 years, and the factors that affected the therapeutic responses were analyzed, including the type of vitiligo, stable time and duration of the disease, and location of the lesions. We primarily focused on the size and ratio of the vitiligo lesion areas.

## 2 METHODS

### 2.1 Ethics statement

This retrospective review was approved by the Medical Ethics Committee of Third People's Hospital of Hangzhou. Electronic medical records of all patients who underwent CMT at the Department of Dermatology, Third People's Hospital of Hangzhou, between June 2009 and June 2018 were retrieved. The patients were followed up until June 2020. The clinical information of all patients was available for at least 12 months after the procedure. The inclusion criterion for CMT was stable vitiligo (static lesions for at least 6 months and absent Koebner phenomenon) failing to respond to conventional topical and systemic therapies. Pregnant patients, patients with a history of keloid formation, and those with bleeding diathesis were excluded. No washout period was required for the patients receiving topical or systemic medications.

Clinical and demographic data, characteristics, and types of vitiligo were obtained preoperatively. Routine clinical photographs were obtained for all patients before CMT and at each follow‐up visit. To compute the target area treatment ratio of varied lesions, we used target area treatment ratio = CMT area/target leukoplakia lesion area × 100%. Physicians evaluated the percentage of repigmentation at 3, 6, 9, and 12 months and yearly thereafter. Repigmentation of >90%, 50%–89%, 20%–49%, and <20% was graded as excellent, good, fair, and poor, respectively.

### 2.2 Cell culture

We took samples from the abdomens or buttocks of patients with normally pigmented skin and placed them under vacuum at 40 KPa for 60–90 min to produce blisters with diameters of 8 mm. Four blisters were prepared per patient. The blister roofs were cut and placed in a vial containing calcium‐free Hank's solution. The specimen was cleaned twice with calcium‐free Hanks solution (D‐Hanks solution) and incubated in 0.25% trypsin solution followed by 0.02% ethylenediaminetetraacetic acid (EDTA) solution (both incubated at 37°C for 10 min). The cells were then separated from the epidermis using a stereomicroscope. After centrifuging the cell suspension, they were cultured in the bottle with 16 HU medium (F12 medium containing 20 ng/mL basic fibroblast growth factor, 20 μg/mL 3‐isobutyl‐1‐methylxanthine, 10 ng/mL cholera toxin, 50 μg/mL gentamicin, and 10% fetal bovine serum). The cells were cultured in an automatic carbon dioxide regulation incubator. After culturing for 3 days, genogenes were added to the culture solution (100 μg/mL) to remove contaminated cells. After fusion of the primary culture, the melanocytes were isolated with 0.125% trypsin/0.01 EDTA solution, diluted to a ratio of 1:2–1:3, and inoculated into bottles for subculturing.

### 2.3 Preoperative preparation

For the preparation of the recipient's transplantation site local anesthetic (lidocaine) cream was applied 2–4 h before transplantation. The recipient area was washed with 70% alcohol, and the epidermis was removed using a superpulsed CO_2_ laser (30–50 Hz, 225 mJ).

### 2.4 Cell transplantation

The transplant concentration of melanocytes was 600–1000/mm^2^.

### 2.5 Statistical analyses

Each variable was compared among the cohorts using analysis of variance or Kruskal–Wallis tests for continuous variables and Pearson's *χ* ^2^ or Fisher's exact test for categorical variables. When these unadjusted time‐to‐event comparisons were statistically significant, Cox proportional hazards regression models were used to estimate hazard ratios and 95% confidence intervals for pairwise comparisons, adjusting for potential confounding factors. Two‐factor regression analysis was used to detect differences in efficacy‐related factors among the different transplantation standards. Multivariate logistic regression analysis was used to determine the importance of various variables in transplant results. The level of statistical significance was set at *P* < 0.05, and all analyses were performed using SPSS version 17.

## 3 RESULTS

### 3.1 Patient characteristics

This analysis included 491 patients with long‐term follow‐up data (12–120 months, mean 43 months, median 36 months). Table 1 illustrates the demographic and vitiligo characteristics at baseline. The duration of vitiligo ranged from 1 to 30 years, with a mean duration of 6.5 years. A total of 176 (35.8%) patients had segmental involvement and 315 (64.2%) had NSV. The maximum area operated on in one lesion was over 200 cm^2^ and the minimum was 30 cm^2^. In total, 123 (5.4%) patients experienced vitiligo recurrence after 12 years. The face and neck were the two most common transplanted sites, followed by the back of the hands.

**TABLE 1:** Baseline characteristics of participants.

Characteristic	All patients (*N* = 491)	Segmental (*N* = 176)	Nonsegmental (*N* = 315)	*P* value (between two groups)
Age, mean ± SD, years	24.3 ± 11.1	22.7 ± 9.5	25.2 ± 11.8	>0.05
Age at disease onset, mean ± SD, years	18.5 ± 7.5	16.4 ± 7.1	19.4 ± 8.3	>0.05
Disease duration, mean, months	79.9	63.1	89.4	<0.05 (*)
Female, *n* (%)	286 (58.2)	101 (57.4)	185 (58.7)	>0.05(#)
Male, *n* (%)	205 (41.8)	75 (42.6)	130 (41.3)	
BSA (cm^2^), mean ± SD	55 ± 30	53.5 ± 26.8	55.8 ± 32.7	>0.05
Total area (100 cm^2^), mean	6.7	1.84	9.4	<0.05 (*)
Stable time, minutes	24.3	30.8	20.8	<0.05 (*)

*Note*: These results represent *P* values of the null hypothesis (*P < 0.05). #Reprents female and male’s *P* values both have no statistical significance.

Abbreviations: BSA, Body surface area; SD, standard deviation.

These 491 patients were treated with CMT with sizes ranging from 30 to 640 cm^2^. Among these lesions, excellent, good, fair, and poor repigmentation was observed in 293 (59.7%), 90 (18.4%), 63 (12.8%), and 45 (9.2%) lesions, respectively, and the mean extent of repigmentation was 75.3 ± 30.4, with a total effective rate (the repigmentation ≥50%) of 78%. Significant repigmentation was observed in most cases at 1–3 months after transplantation. All patients were followed up for at least 3 months, with a maximum of 12 years.

### 3.2 Regression analysis of clinical features related to repigmentation efficacy

A first‐step multivariate logistic linear regression analysis was used to determine the importance of various variables on repigmentation results after transplantation and analyze the relevance of different clinical features to treatment efficacy. The type, age, and stability of vitiligo play key roles in predicting efficacy (*P* < 0.01). The feature of area in transplantation lesion area, lesion distribution, and total body area were all weakly correlated with efficacy (*P* = 0.189). Among these areas, the distribution of future lesions was decisive for the results (*P* < 0.01; Table 2).

**TABLE 2:** Multiple linear regression analysis of clinical features.

	Non‐standard		Standard coefficient	*t*	Significance
	*B*	*SE*			
Type	−13.178	2.893	−0.208	−4.566	0.000
Stable	0.104	0.040	0.135	2.626	0.009
Duration	−0.007	0.021	−0.017	−0.325	0.745
Area	0.057	0.043	0.058	1.316	0.189
Constant	91.758	5.422		16.923	0.000
Age	−0.381	0.122	−0.139	−3.115	0.002

*Note*: This table shows the regression results on the impact of type and stable vitiligo on efficacy.

Abbreviation: SE, Standard Error.

Efficacy, including excellent and good repigmentation binomial logistic regression analyses, was used to determine the importance of various variables. The type and stability of vitiligo also play key roles in its efficacy (*P <* 0.05; Tables 3 and 4). Although vitiligo duration has been identified as an unrelated factor associated with successful outcomes, the area feature showed a slightly stronger correlation with efficacy (*P* = 0.1; Table 4).

**TABLE 3:** Binomial logistic regression analysis of excellent repigmentation.

	*B*	*SE*	Wald	*df*	Significance	Exp(*B*)
Type	−1.192	0.340	12.272	1	0.000	0.304
Stable	0.020	0.009	5.544	1	0.019	1.021
Duration	0.000	0.002	0.264	1	0.607	0.999
Area	0.003	0.004	0.610	1	0.435	1.003
Constant	3.263	0.665	24.043	1	0.000	26.123

*Note*: This table shows the logistic regression results on the impact of type and stable vitiligo on excellent repigmentation.

Abbreviation: SE, Standard Error.

**TABLE 4:** Binomial logistic regression analysis of good repigmentation.

	*B*	*SE*	Wald	*df*	Significance	Exp(*B*)
Type	−0.667	0.210	10.061	1	0.002	0.513
Stable	0.009	0.004	5.101	1	0.024	1.009
Duration	0.001	0.001	0.287	1	0.592	1.001
Area	0.005	0.003	2.705	1	0.100	1.005
Constant	0.950	0.397	5.723	1	0.017	2.586

*Note*: This table shows the logistic regression results on the impact of type and stable vitiligo on good repigmentation.

Abbreviation: SE, Standard Error.

### 3.3 Transplantation relationship among the stable/type/area of vitiligo

Among the 201 lesions with stable vitiligo for 1 year, 99 lesions had >90% repigmentation, accounting for 49.2%, and 164 lesions of the 201 lesions had >50% repigmentation, accounting for 81.5%.

The average degree of pigmentation was 69.5%. Repigmentation up to 90% or more was seen in 116 out of 159 lesions with vitiligo stable ≥2 years, accounting for 73%, and repigmentation over 50% or more was seen in 142 out of 159 lesions with NSV, accounting for 89.3%. The average extent of repigmentation was 83.3%. Repigmentation of up to 90% or 50% between SV and NSV showed statistically significant differences (*x* ^2^ = 25.716, *P <* 0.0001). The longer the stabilization time, the better, and efficacy between the two groups was significantly different (*P* < 0.05; Table 5).

**TABLE 5:** Multiple indicators analysis of the triple repigmentation rate.

	Cases (*n*)	Excellent repigmentation (*n*, %)	Good repigmentation (*n*, %)	Average repigmentation
Stable time, year				
<1	201	99 (49.2)	164 (81.5)	69.5
≥1	290	194 (66.9)	249 (85.8)	79.3
≥2	159	116 (73)	142 (89.3)	83.3
*P* value		<0.05 (*)	<0.05 (*)	<0.05 (*)
Lesion type				
Segmental	176	123 (69.9)	164 (93.2)	84.4
Nonsegmental	315	170 (54)	219 (69.5)	70.1
*P* value		<0.05 (*)	<0.05 (*)	4.86E−07
Lesion distribution				
Forehead	14	8 (57.1)	11 (78.6)	67.2
Face and neck	155	99 (63.9)	136 (87.7)	78.9
Trunk	126	74 (58.7)	102 (81)	74
Arms and legs	39	23 (59)	35 (89.8)	76.4
Hands and feet	34	15 (44.4)	25 (73.5)	65.5
*P* value		<0.05 (*)	<0.05 (*)	1.6518E−05

*Note*: These results represent *P* values of the null hypothesis (*P < 0.05).

The study area was divided into five main parts for statistical analyses. Through a comparative analysis of the recoloration rates of the five skin areas, significant differences were observed in each region (*x* ^2^ = 7.16, *P <* 0.05). The excellent repigmentation rate of the face and neck was the highest at 63.9%, whereas that of the hands and feet was the lowest at 44.4%. The highest repigmentation rate was observed in the arms and legs (89.8%) and the lowest rate of 73.5% was observed in the hands and feet.

**FIGURE 1:**
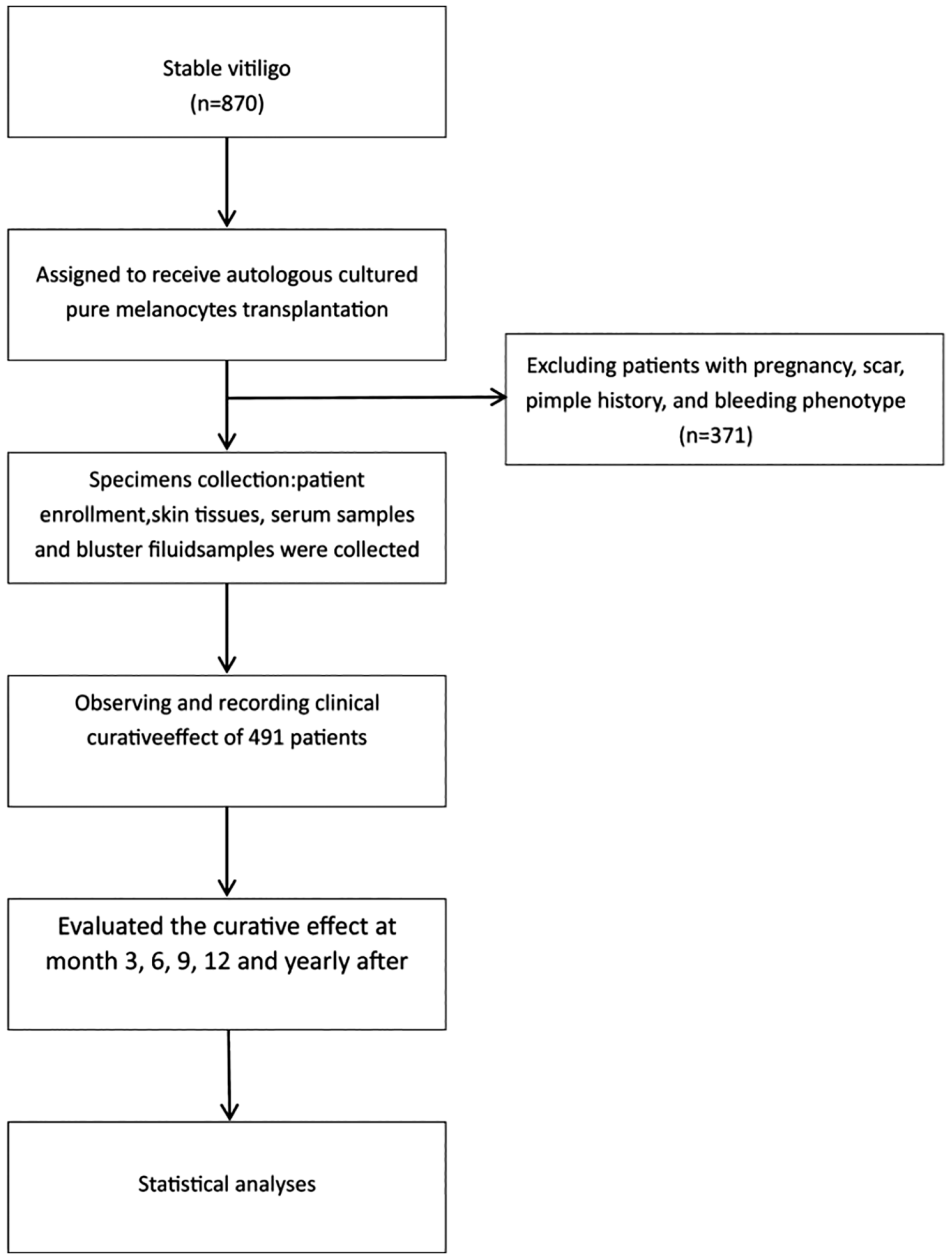
Consort flow diagram of random controlled trials.

In Figure 1, the horizontal axis represents the transplanted area, and the vertical axis ratios represent the transplanted area/target lesion area. The number of cases represents the number of independent transplantations performed.

### 3.4 Relationship between the outcome of transplantation site with different areas and ratios

The subdivisions and curative effects of the regions, the effective and recovery rates of the different regions, and the average repigmentation rate of multiple areas were significantly different.

**FIGURE 2:**
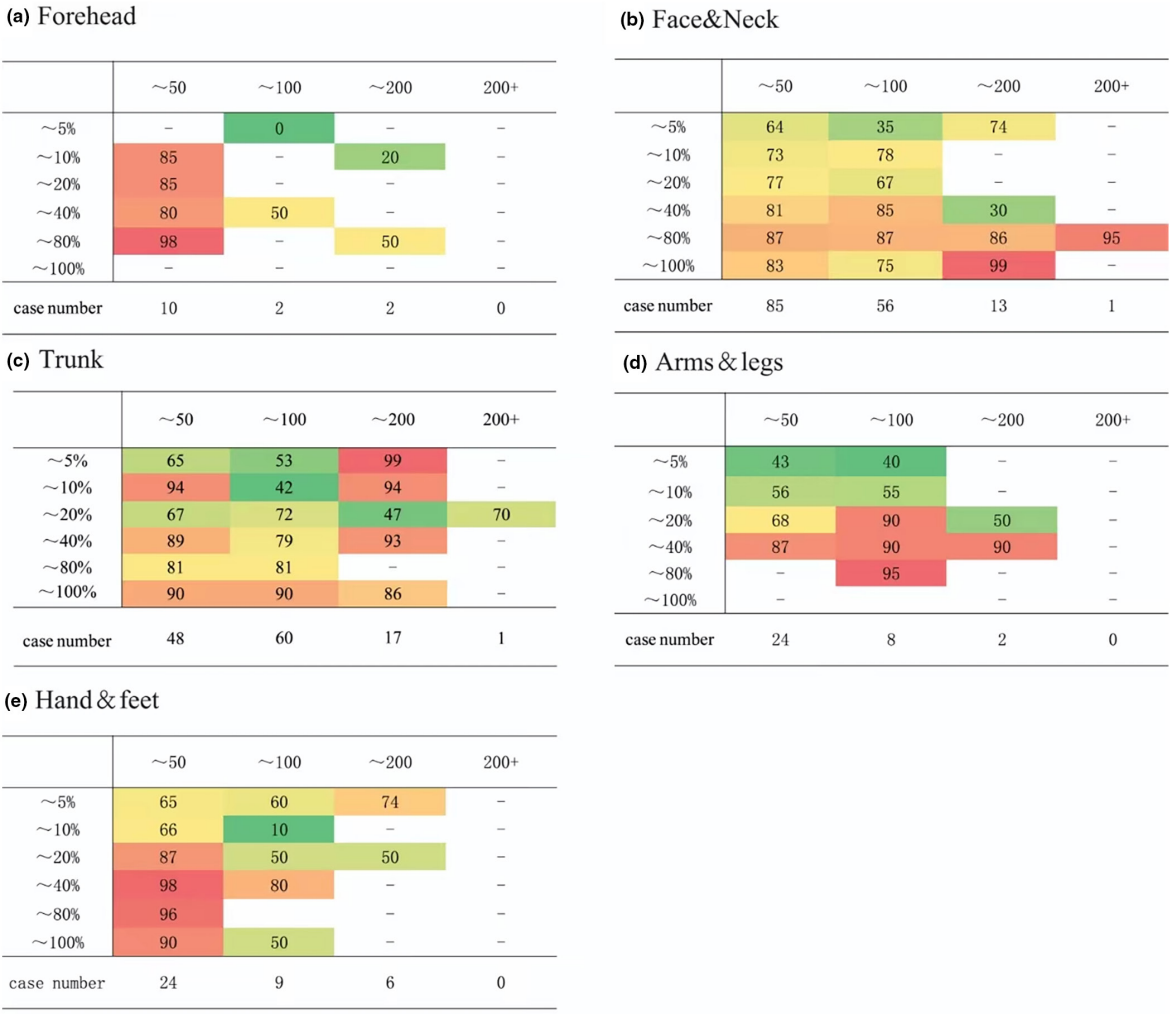
The difference in the efficacy of the treatment area of the transplant site with different areas and ratios. (a) The ratio and case number of forehead transplantation. (b) The ratio and case number of face and neck transplantation. (c) The ratio and case number of trunk transplantation. (d) The ratio and case number of arms and legs transplantation. (e) The ratio and case number of hands and feet transplantation.

We further analyzed the difference between the total transplanted area and the proportion of the area on the recoloration rate, and we used these as the horizontal and vertical quadrants to analyze the transplanted repigmentation rate. The results showed that, regardless of the part involved, large‐scale treatment was not necessarily effective. For different skin areas, some are suitable for large‐area and large‐ratio transplantations, whereas others are suitable for small‐ and medium‐area transplantations. However, the efficiency of large‐ratio transplantation was much higher than that of low‐ratio transplantation, and the results were linearly related (Figure 2).

**FIGURE 3:**
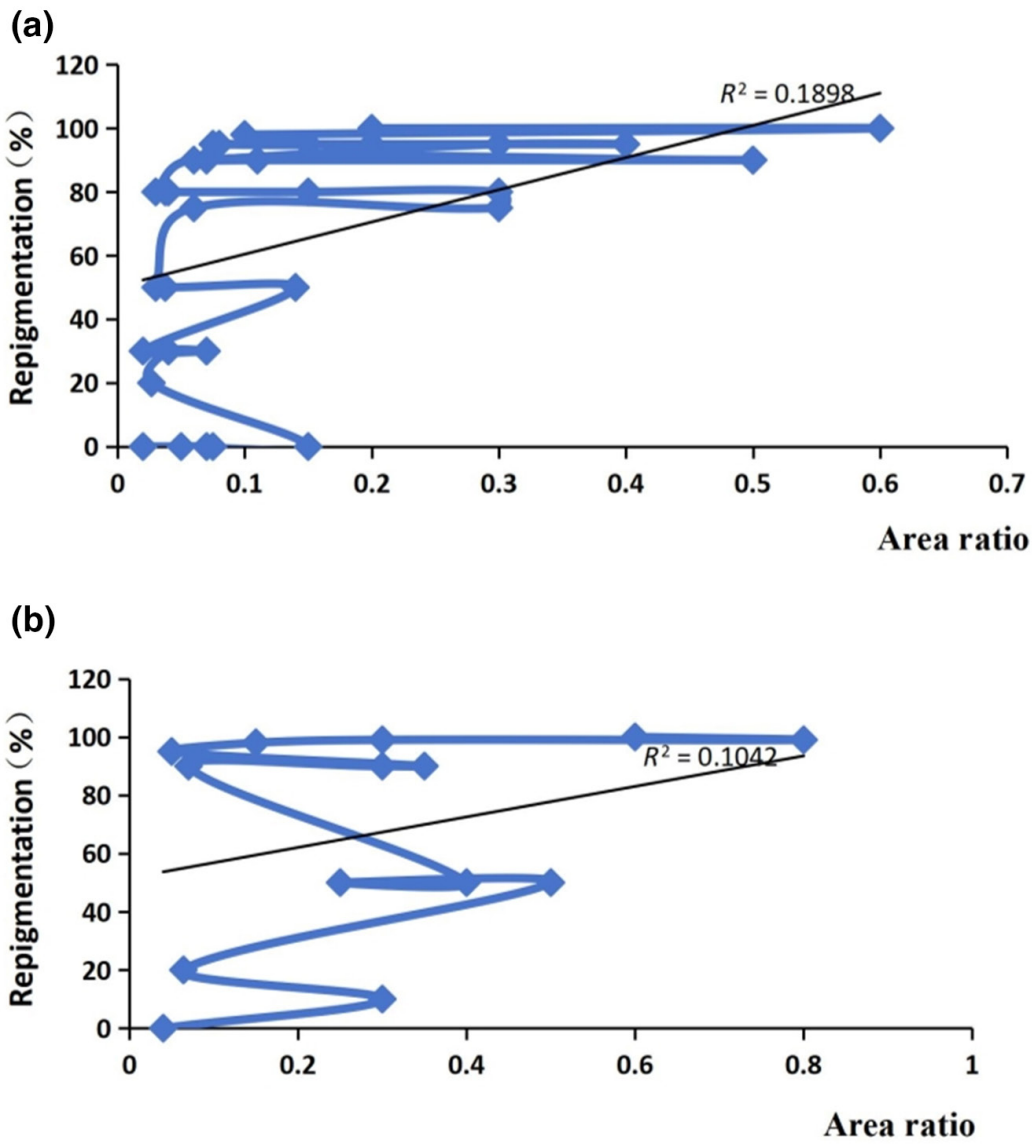
Linear regression analysis of area ratio and repigmentation rate. (a) The different repigmentation ratio of hands and feet. (b) The different repigmentation ratio of forehead.

The repigmentation rate of the face and neck area (50/100 cm^2^) in the first two groups was linearly correlated with the area ratio *r* = 0.83/0.53, and the area of the front two groups of the trunk (50/100 cm^2^) was linearly correlated with the area ratio *r* = 0.41/0.85. In the first three groups of arms and legs (50/100/200 cm^2^), the repigmentation rate was linearly correlated with the area ratio, with *r* values of 0.98, 0.75, and 0.69, respectively (Figure 2). The forehead and limbs were suitable for small areas and large proportions of transplantation (Figure 3). The face, neck, and trunk limbs could be transplanted over a large area in a large proportion, and the effect was better. Regardless of the skin area, the effect of the transplant was much better than that of small coverage.

### 3.5 Adverse event

Three patients developed local infections, which were attenuated and eliminated by oral antibiotic treatment. No other adverse events, such as hypersensitivity and scar hyperplasia, occurred after transplantation.

## 4 DISCUSSION

**FIGURE 4:**
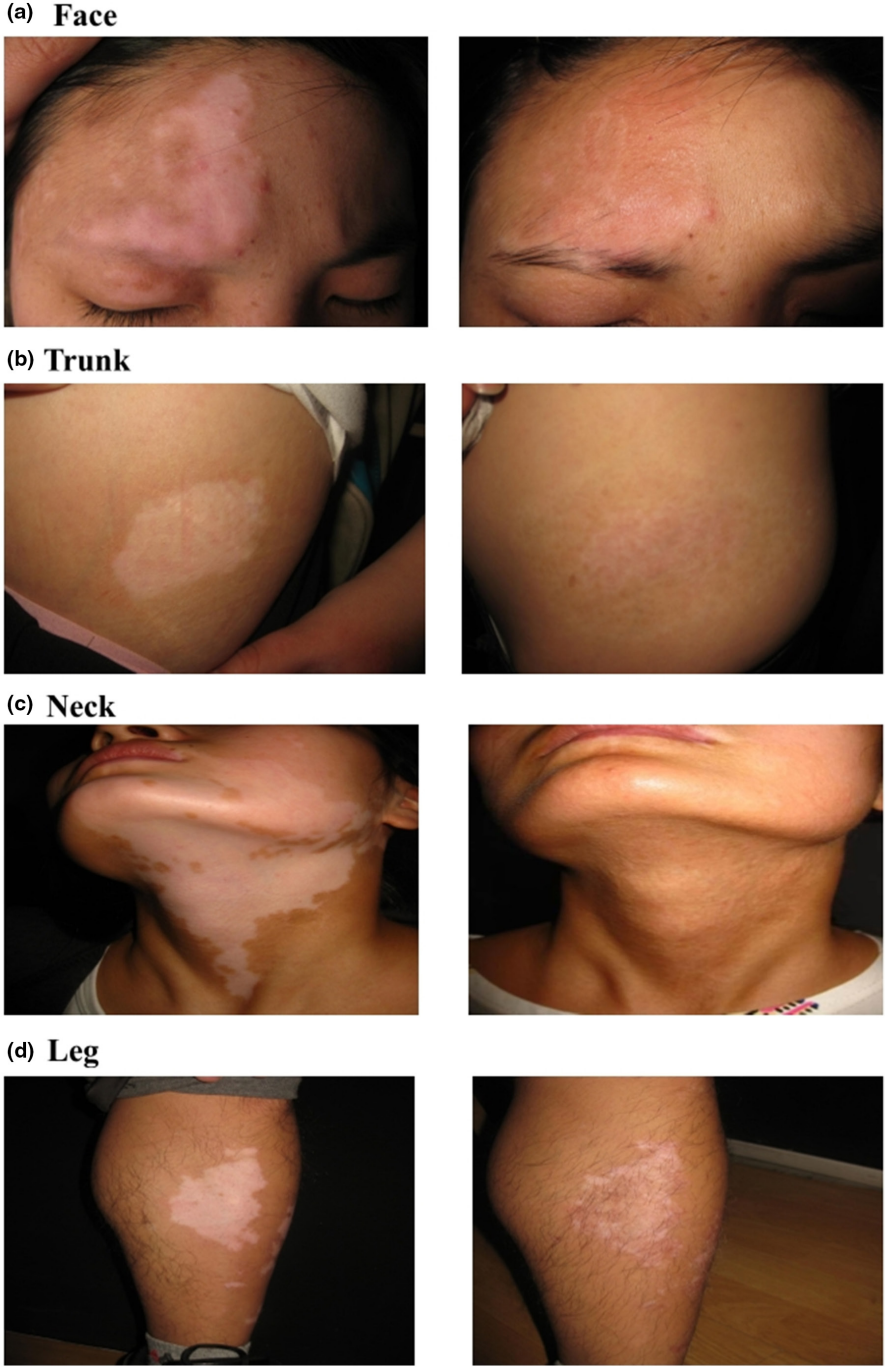
The recovery of autologous melanocyte transplantation at various sites. (a) The recovery rate of the face. (b) The recovery rate of the trunk. (c) The recovery rate of the neck. (d) The recovery rate of the leg.

This study evaluated the correlation between the type and stability of vitiligo and the efficacy of CMT treatment. Transplantation outcomes can be influenced by the location of skin lesions.^19,21,23,24^ The fingers and elbows are the most difficult areas for repigmentation. The trunk, arms, and legs (not including the elbows and knees) respond best.^19,21,23,24^ In the current study, skin lesions located on the head, neck, and trunk showed better outcomes than those located elsewhere (Figure 4).

CMT is an effective method for SV and NSV in the stable stage. Patients with SV are more susceptible to CMT treatment because the leukoplakia caused by SV is easier to stabilize. In fact, although patients with NSV have a long onset cycle of the white spot site in multiple and large areas, after white spot stabilization, they tend to respond to CMT. Due to differences in pathogenesis, cell heterogeneity, and skin microenvironment at the transplant recipient site, recoloration after transplantation of melanocytes will also differ, and this repigmentation rate has been demonstrated in different studies. SV lesions mainly occur on the extremities, such as the hands, face, and neck, and their boundaries with the surrounding skin are relatively clear. Generally, local topical hormone or calcineurin inhibitor treatment combined with phototherapy is used, and its effect on the extremities is far worse than that on the face and neck. The common NSV appears mainly in the face, neck, back of the hand, extremities, and other parts that can affect any part of the body. The treatments of small‐area disease (<3 BSA) are the same as those of SV, large‐area diseases that require systemic oral drug treatment.^25,26^

According to a recent study, vitiligo is induced by activated melanocyte antigen‐specific CD8+ T cells, which drive cytotoxicity and disease pathogenesis. Interferon‐gamma (IFN‐γ) mediates recruitment of autoreactive CD8+ T cells to melanocytes through IFN‐γ‐induced chemokines such as C‐X‐C motif chemokine ligands CXCL 9 and CXCL10.^27–29^ Moreover, anatomically distinct human dermal fibroblasts exhibit intrinsic differences in the expression of chemokines in response to IFN‐γ.^30^ Thus, the skin location has become a key clinical factor that influences efficacy.

Here, we analyzed the relationship between the results of the different areas and the proportions of graft sites. We could clearly observe the importance of the transplant ratio on treatment efficacy. Selecting the appropriate transplant area for different areas and increasing the transplant ratio will help improve transplant efficacy. It has been emphasized that the donor‐to‐recipient ratio is key to the CMT transplantation technique, as CMT can have higher ratios, from 1:1 to 1:100.^13,31^ However, the choice of the patient site and graft ratio may be more important when considering stable cell culture and transplantation techniques.

Consistent with the findings of previous studies, SV achieved better outcomes than NSV. Etiological differences could explain variations in transplantation results. According to Olsen's many studies, stable time is the most critical selection factor for vitiligo, and only stable vitiligo can be transplanted regardless of the size and type of refractory vitiligo.^18^ ^,19^ In this study, the length of the stable period influenced the transplantation outcomes. The efficacy rate in stable time >12 months showed a significant difference when compared to that in <12 months. This may be due to the methodology used for cultivating epidermal melanocytes in our laboratory. The levels of the C‐X‐C motif chemokine ligands CXCL9 and CXCL10 in blister fluid samples were significantly lower in stable patients than in active participants.^32^ Changes in the CXCL9 and CXCL10 levels may be associated with the efficacy of CMT.

## 5 CONCLUSION

Collectively, our findings suggest that CMT is a viable alternative for patients with vitiligo refractory to medical therapy. Various factors, such as disease stability duration, vitiligo type, and lesion site, should be carefully evaluated before use, as these factors further influence postoperative pigmentation. In particular, the selection of the transplant area and transplant proportion will greatly impact the success and recoloration rates of the transplant and affect efficacy and satisfaction. We plan to investigate further whether anti‐inflammatory or corticosteroids/systemic steroids treatments increase the efficacy of CMT.

## CONFLICT OF INTEREST STATEMENT

None declared.
